# Breast self-examination practice and associated factors among female healthcare workers in West Shoa Zone, Western Ethiopia 2019: a cross-sectional study

**DOI:** 10.1186/s13104-019-4676-3

**Published:** 2019-09-30

**Authors:** Seifadin Ahmed Shallo, Jibril Dori Boru

**Affiliations:** 1 Department of Public Health, Ambo University, P.O.box 19, Ambo, Ethiopia; 2 Ambo University Referral Hospital, Ambo, Ethiopia

**Keywords:** Breast self-examination practice, Female health workers, West Shoa

## Abstract

**Objective:**

Breast cancer is the leading cause of death among reproductive-age women worldwide and the second leading cause of death among women in Ethiopia. Regular breast self-examination is the most cost-effective methods for early detection of breast cancer. Despite this fact, breast self-examination was low among women in the general population and it was not well documented among health care workers. Therefore, this study intended to assess the magnitude of breast self-examination practice and associated factors among female healthcare workers in public health facility in West Shoa Zone, Ethiopia. Institutional based cross-sectional study was conducted among 379 female healthcare workers. The study participants were recruited by lottery method. Data were collected from March to April 2019. Data were entered into Epi data version 4.5 and analyzed using SPSS version 25. Bivariate and multiple logistic regressions analysis were done. With 95% CI, the level of significance was decided at P-value ≤ 0.05.

**Results:**

The magnitude of regular breast self-examination practice was 32.6%. Educational levels, breast cancer awareness, knowledge towards breast self-examination were predictors of breast self-examination. Regular awareness creation forum on breast self-examination technique, risk factors, and related matters should be facilitated so that all female health care workers will be reminded.

## Introduction

### Background

Breast cancer is the leading cause of cancer mortality worldwide. In 2017, an estimated 252,710 new cases of invasive breast cancer were diagnosed among women and approximately 40,610 women were expected to die from breast cancer [1]. It’s also the most frequently diagnosed cancer in women, with an estimated 1.7 million new cases and 521,900 deaths [2].

Since breast cancer is serious, an important factor in the prognosis of breast cancer is early detection of the disease [3]. Breast self-examination (BSE) is one of the simple, quick, and cost-free procedures for early detection of breast cancer among women [4]. In developing countries, breast self-examination is the recommended method because it is easy, convenient, private, safe and doesn’t require equipment [5, 6]. Several studies proposed it because there is a difference among women who practice BSE and those not [7].

Early detection of breast cancer plays an important role in decreasing its morbidity and mortality. Studies from some African Countries indicated that the duration of onset of breast cancer to care seeking period is more than 1 year and implies that improving timely detection of breast cancer among women in SSA could have a great impact on stage at diagnosis, and gives opportunity for improved prognoses and treatment options. Most of the early breast tumors are self-discovered and the majority of early detection is by BSE, and 80% may be detected by expert professionals [8, 9]. Evidence indicates that lack of time, lack of self-confidence in their ability to perform the technique correctly, fear of possible discovery of a lump, and embarrassment associated with manipulation of the breast have been cited as reasons for not practicing BSE [10].

Early detection and diagnosis rate of breast cancer among Ethiopia women is considerably low when compared to women in Western countries. This fact put the Ethiopian women’s to be diagnosed at the late stage of the disease. Therefore, many women miss early detection and treatment opportunities due to the lack of information and knowledge of breast cancer, as well as cancer screening skills.

The magnitude of BSE varies among different segments of females. For instance, a study conducted among undergraduate university student in Addis Ababa indicated 21%, study in Debra Birhan indicated 28%, while the study among female health care workers indicated somehow greater prevalence. For example study among health extension workers in Gojam, Northern Ethiopia shows 37% and 32% in Debre tabor. Even though the finding among health care workers seems relatively higher when compared to other groups of females, it has two main limitations. The first is different studies used different tools for assessing the BSE practice and this curbs comparison of the studies. Secondly, even with the currently existing evidence, the difference in the prevalence of BSE among female health care workers and other female groups is not as it should be [11, 12].

Therefore, this study attempted to assess the BSE practice and associated factors using input from different works of literature and applying the standard and correct definition of BSE.

## Main text

### Methods

This study was conducted in seven Public hospitals found in west Shoa Zone Oromia regional state, Ethiopia using institutional-based cross-sectional study design. Data were collected from March to April 2019.

The sample size was determined using single population proportion formula with the assumption of marginal error of 5%, 5% of non-response rate, 95% confidence level and the prevalence of the breast self-examination practice to be 37% from the study conducted in East Gojjam, North Ethiopia [13].

Since the sample was drawn from a finite population, the correction formula was applied. Finally, the sample size of 379 was determined. The calculated sample size was proportionally allocated to each Hospital based on the number of female health care workers in the hospital. After proportionally assigning sample size to each hospital, a simple random sampling technique was applied to select study participants.

#### Inclusion and exclusion criteria

All-female healthcare workers who were actively on job during data collection at each selected hospitals were included.

#### Data collection tools and techniques

Data was collected using a self- administered questionnaire. The questionnaire was developed in the English language after reviewing and extracting from different pieces of literature developed for the same purpose. For measuring knowledge towards BSE, there were 10 questions developed. Answering a correct answer will result in scoring a mark and loosing will attract zero scores. Accordingly, the final total mark will be added up out of ten and graded for the decision of knowledge level.

To measure attitude towards BSE, Likert scale based items were prepared (total of ten questions). The scales reached from strongly agree to strongly disagree. To assess the internal consistency of the items, Cronbach alpha was assessed and it was 0.87 indicating good internal consistency of the items.

### Operational definitions

Good practice of breast self-examination: Those who performed breast self-examination practice a week after each menses by their palm and middle three fingers otherwise called poor practice.

Good knowledge: Participant those who answered greater than 75% of the 10 knowledge questions towards breast self-examination.

Average knowledge: Participants who answered 50–75% of knowledge questions toward breast self-examination.

Poor knowledge: Participants who answered less than 50% of knowledge questions toward breast self-examination.

Favorable attitudes: Participants who scored points equal to or greater than mean score of breast self-examination related attitude questions as measured by Likert scale.

Unfavorable attitude: Participants who scored points less than the mean score of attitude questions [11, 14].

#### Data management and analysis

The collected data were checked visually for completeness, then coded and entered into Epi data version 4.5 statistical packages. Descriptive analysis was computed. To assess the association between dependent and independent variables by controlling for confounders, first binary logistic regression was run and variables with P-value ≤0.25 and the variables which are known to have an association with dependent variables from reviewed literature were selected for Multiple logistic regression analysis. Statistical significance was declared at P-value < 0.05 with 95% confidence interval (CI).

#### Data quality control

To ensure the data quality of our study the following measures were taken:

- The questionnaire was developed by reviewing relevant pieces of literature on the subject.
- to ensure reliability.
- The questionnaire was pre-tested and modified where necessary.
- One day training was given for data collectors and supervisors.

##### Dependent variable

- Breast self-examination practice (BSE).

#### Ethical consideration

Ethical clearance was obtained from the Ethical Review Committee of the College of Medicine and Health Sciences, Debra Markos University. During the fieldwork, the objective of the study was clearly explained for the study participants, the confidentiality of the data to be collected and the right not to participate was also assured. Before starting the data collection process, written consent was taken from each respondent after they read and signed the consent form.

### Result

#### Socio-demographic characteristics of the study participants

A total of 340 female healthcare workers were responded to the distributed questionnaires, making a response rate of 89.7%. The mean age of the respondents was 28.0 ± 4.8 SD which was ranging from 19 to 43. About 187 (55.0%) was married. Out of the total participants, about 198 (58.2%) were nurse professionals and more than two thirds (232) of the participants were degree holder followed by diploma 71 (20.9%) (Table 1).

**Table 1:** Socio-demographic characteristics of the female health care workers in West Shewa zone public hospitals, Western, Ethiopia 2019

Characteristics	Frequency	Percentage
Age		
19–25	141	41.5
26–30	141	41.5
≥ 31	58	17.0
Marital status		
Single	153	45.0
Married	187	55.0
Ethnicity		
Oromo	276	81.2
Amhara	48	14.1
Others	16	4.7
Religion		
Orthodox	138	40.5
Protestant	177	52.1
Muslims	23	6.8
Others	2	0.6
Profession		
Nurse	198	58.2
Midwife	57	16.8
Pharmacy	21	6.2
Laboratory	18	5.3
Medical doctors	31	9.1
Others	15	4.4
Educational level		
Diploma	71	20.9
Degree	232	68.2
Masters	37	10.9
Work experience		
< 5	174	51.2
≥ 5	166	48.8
Ward units		
Inpatient service	240	70.6
Outpatient service	100	29.4

#### Knowledge towards breast self -examination and breast cancer

**Fig. 1:**
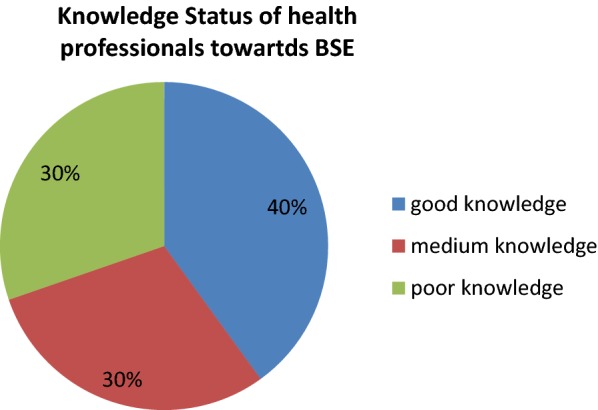
Knowledge towards breast self-examination and breast cancer among female health care workers at west Shoa zone hospitals, Oromia region, Western Ethiopia, 2019

The majority of 227 (66.8%) the study participants know the screening methods of breast cancers. The commonly mentioned breast self-screening techniques were: mammography (5.0%), clinical breast self-examination (7.9%), and 23.2% mentioned breast self-examination. About 106 (31.2%) stated the three screening methods of breast cancer. Out of the total participants, 228 (67.1%) ever heard about breast self-examination. The main source of information of the breast self-examination was the knowledge they received during the class lectures in the University or College which accounts for 132 (38.8%) (Fig. 1).

#### The attitude of the study participants towards BSE practice

The attitude mean score of the study participants was calculated and used as a cutoff point for classifying attitude towards BSE as favorable or not. Accordingly, the mean attitude score was 30.0 ± 4.5 SD. More than half of the participants 202 (59.4%) had a favorable attitude towards breast self-examination.

#### Factors associated with breast self examination

In bi-variate logistic regression three variables i.e. level of education, work experience, and knowledge of BSE have association with breast self examination, while in multiple logistic regression, level of education, attitude towards BSE, and knowledge of BSE were independent predictors of breast self examination practice (Table 2).

**Table 2:** Multiple logistic regression analysis results for the association between the practice of breast self-examination and independent variables among female healthcare workers, West Shoa Zone Hospitals, Oromia region, Western Ethiopia, 2019

Variables	BSE practice		COR with 95% CI	COR with 95% CI	P-value
	Good	Poor			
Educational level					
Diploma	23	48	0.01 (0.002, 0.103)	0.03 (0.004, 0.260)	0.001*
Degree	118	114	0.03 (0.004, 0.213)	0.08 (0.008, 0.490)	0.008*
Masters	36	1	Ref	Ref	
Work experience					
< 5	100	74	1.60 (1.02, 2.39)	0.90 (0.56, 1.65)	0.8
≥ 5	77	89	Ref	Ref	
Ward unit					
Inpatient service	133	107	1.60 (0.98, 2.53)	1.13 (0.64, 1.98)	0.7
Outpatient service	44	56	Ref	Ref	
Do you taught BSE to the client?					
Yes	19	60	0.25 (0.16, 0.39)	0.40 (0.23, 0.75)	0.004*
No	97	47	Ref	Ref	
Do you know people with Breast cancer					
Yes	118	80	Ref	Ref	
No	59	83	0.50 (0.3, 0.75)	0.90 (0.45, 1.57)	0.6
Knowledge about BSE					
Good	110	26	13.20 (7.09, 24.56)	6.70 (3.24, 14.04)	< 0.001*
Average	42	59	2.20 (1.22, 4.04)	1.80 (0.96, 3.39)	0.067
Poor	25	78	Ref	Ref	
Attitudes towards BSE					
Favorable	112	90	0.70 (0.46, 1.105)	0.60 (0.53, 0.9)	0.047*
Unfavorable	65	73	Ref		

*AOR* adjusted odd ratio, *COR* crude odds ratio

* Significantly associated

### Discussion

In this study, the prevalence of the correct and regular practice of breast self-examination was low (32.6%). This finding is comparable with study conducted among midwifes and Nurses in Turkey and lower than finding from Eritrea (60.6%) [15, 16]. But its higher than the finding in the study conducted in West Gojjam, of Amhara region (14.4%) and finding from Egypt among nurses (18.8%) [13, 17]. The differences could be due to the difference in the study period and study area.

This study indicated, as the educational level of the females’ increase, the likely hood of BSE practices also increase. Compared to those females’ in diploma level, degree holders were 12 times more likely to practice BSE correctly and regularly. This finding is consistent with a study conducted in Western Ethiopia Female health care workers who don’t teach BSE to their clients during routine clinical activities were more than 50% less likely to practice breast self-examination 0.42 (AOR = 0.42, 95% CI 0.23, 0.75) (P-value 0.004) compared their counterparts.

This study also showed that there is a significant association between participants’ level of knowledge about breast cancer and BSE practice. The commonest reasons reported why the females were practicing BSE were: for early detection and seeking treatment and fear of developing breast cancer in the future.

Though they had sufficient knowledge and awareness about BSE, about 27% of the females didn’t practice it because of negligence. This finding is similar with finding from Asmara which was conducted among nurses [16].

In conclusion, the finding of the study indicated that BSE practice was low and it was associated with different factors. There was also a gap in knowledge and awareness among the female health care professionals towards BSE, and even those who had sufficient knowledge were not practicing BSE because of negligence.

Short term training on breast cancer and breast self-examination should be better organized by the hospital administrator.

## Limitation of the study

The self-reported information is subjected to bias specifically to social desirability bias. There was no internationally recognized standardized tool to assess BSE. Besides, since the majority of the existing works of literature were conducted among no health care females, this results in a variation of measurements which limit us comparing the findings of this study with other studies. Also, we didn’t include a qualitative aspect in this study which could have strengthened the finding of a quantitative one.

## Data Availability

All data generated or analyzed during this study were included in this published article and its supplementary information files are available from the corresponding author on reasonable request.

## Abbreviations

ACS: American Cancer Society
AKTH: Aminu Kano Teaching Hospitals
AOR: adjusted odd ratio
BC: breast cancer
BSE: breast self-examination
CBE: clinical breast examination
CI: confidence interval
COR: crude odd ratio
DBU: Debra Berhan University
FHWs: female healthcare workers
HEWS: health extension workers
SHE: Ministry of Science and Higher Education of Ethiopia
SPSS: Statistical Package of Social Science
